# A Case of Pneumothorax Secondary to Marijuana Use Disorder

**DOI:** 10.7759/cureus.26634

**Published:** 2022-07-07

**Authors:** Ashish Jain, Amir Ashiq, Rabia Ahmed, Rahul Prakash Rane, Khandakar M Hussain

**Affiliations:** 1 Internal Medicine, Conemaugh Memorial Medical Center, Johnstown, USA; 2 Internal Medicine, Dow University of Health Sciences, Civil Hospital Karachi, Karachi, PAK

**Keywords:** substance use disorder (sud), primary spontaneous pneumothorax, marijuana use, spontaneous pneumomediastinum (spm), primary pneumothorax

## Abstract

In recent decades, the general tendency has switched from the use of tobacco products to the inhalation of marijuana with or without the addition of tobacco. The majority of existing research on marijuana use focuses on its euphoric effects. Pneumothorax, pneumomediastinum, and subcutaneous emphysema are infrequently described in the medical literature in association with cannabis use. It is a diagnostic and therapeutic challenge because of its infrequency of occurrence. We see a huge diversity of multisystem involvement linked with marijuana smoking, and physicians should be aware of this uncommon clinical presentation, which might be observed more often because of the recent upsurge in its consumption. We describe a case of a 20-year-old male with a chronic history of marijuana use disorder who was found to have non-tension type pneumothorax, pneumomediastinum, and subcutaneous emphysema on chest imaging. The patient was managed conservatively and did not require any surgical intervention.

## Introduction

Marijuana is the most commonly consumed illicit recreational substance in the United States. Growing popular support for marijuana and legislation reforms have led to a substantial rise in its use. The effects of chronic marijuana use on pulmonary health have received less investigation and discussion than those of tobacco. Marijuana use is related to adverse outcomes such as impaired short-term memory, altered judgment, motor incoordination, and paranoia, and cancers of the head, neck, lungs, and gonads have also been documented. Other unusual side effects that have been reported include hemoptysis and myocardial infarction [1]. It is imperative to note that in the past, marijuana was used for a variety of medicinal uses, including as an antiemetic, analgesic, muscle relaxant, and appetite stimulant. As a result of its anti-inflammatory, anxiolytic, hypnotic, and antidepressant properties, marijuana is currently being widely utilized in various therapeutic contexts [2]. Deep inhalation of marijuana accompanied by breath-holding has been linked to pneumomediastinum, pneumothorax, and subcutaneous emphysema [3]. We report a case of a 20-year-old male with a history of marijuana use disorder who was found to have a non-tension pneumothorax, pneumothorax, and subcutaneous emphysema.

## Case presentation

**Figure 1:**
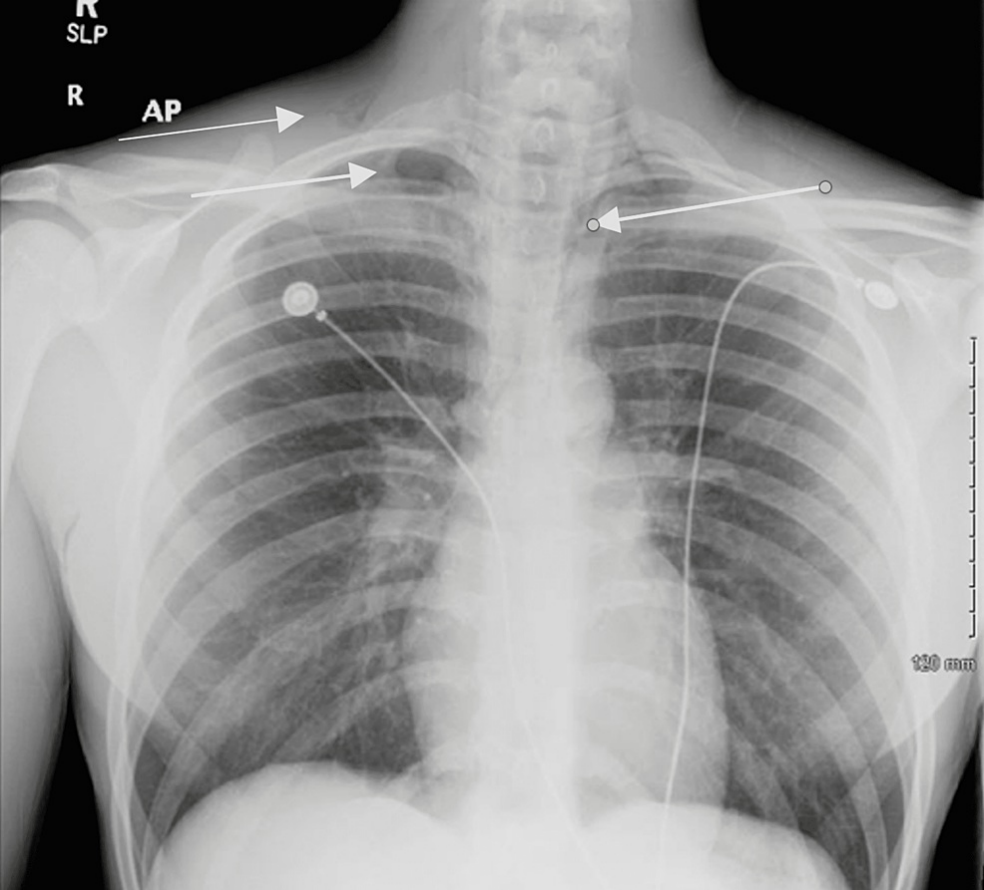
Chest X-ray showing pneumomediastinum with small right apical pneumothorax and subcutaneous air (arrows).

**Figure 2:**
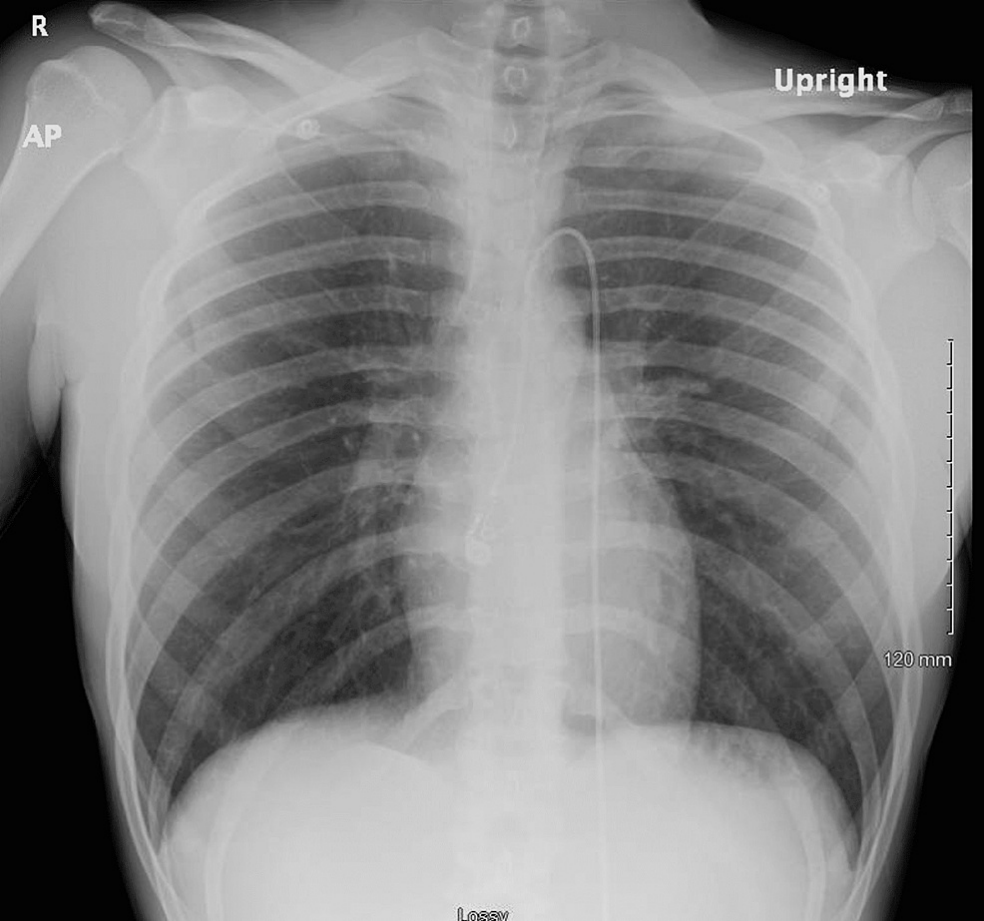
Chest radiograph in the anteroposterior view did not show any evidence of acute or chronic cardiovascular processes.

A 20-year-old man with normal height and weight and a past medical history of chronic inhalational marijuana use disorder associated with secondary cyclic vomiting syndrome presented with progressive, 10 on a scale of 1-10, burning pain in the epigastrium that was associated with nausea and multiple episodes of non-bilious vomiting for the last few hours. An esophagogastroduodenoscopy was performed two months prior to the presentation, which indicated moderate gastritis and was otherwise unremarkable. On presentation, the patient appeared fatigued, although he was alert and oriented to time, place, and person. On physical examination, the patient's heart rate was 98 beats per minute, temperature was 97.7°C, respiratory rate was 18 breaths per minute, blood pressure was 145/80 mmHg, and oxygen saturation was 100% on room air. There were no accompanying rhonchi, wheezes, or crepitations on auscultation of the chest. Laboratory findings were remarkable for creatinine of 2.4 mg/dL, which was elevated from a baseline of 1.2 mg/dL. A chest X-ray revealed pneumomediastinum with small right apical pneumothorax and subcutaneous air (Figure 1). The patient had undergone a chest X-ray in the past, which did not show any evidence of acute or chronic cardiopulmonary processes (Figure 2).

**Figure 3:**
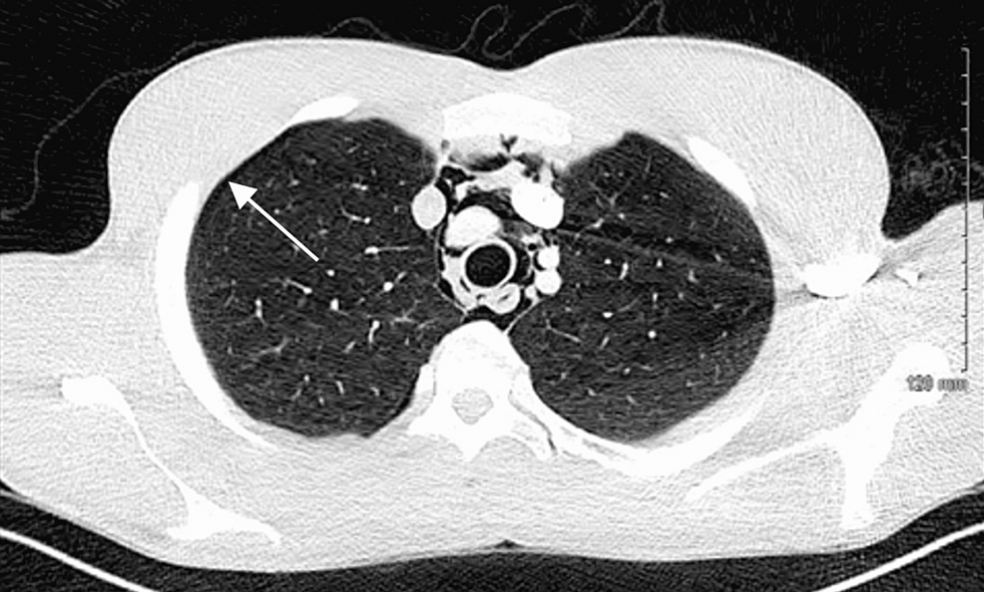
CT of the chest with contrast in the coronal plane showing small right-sided non-tension-type apical pneumothorax.

**Figure 4:**
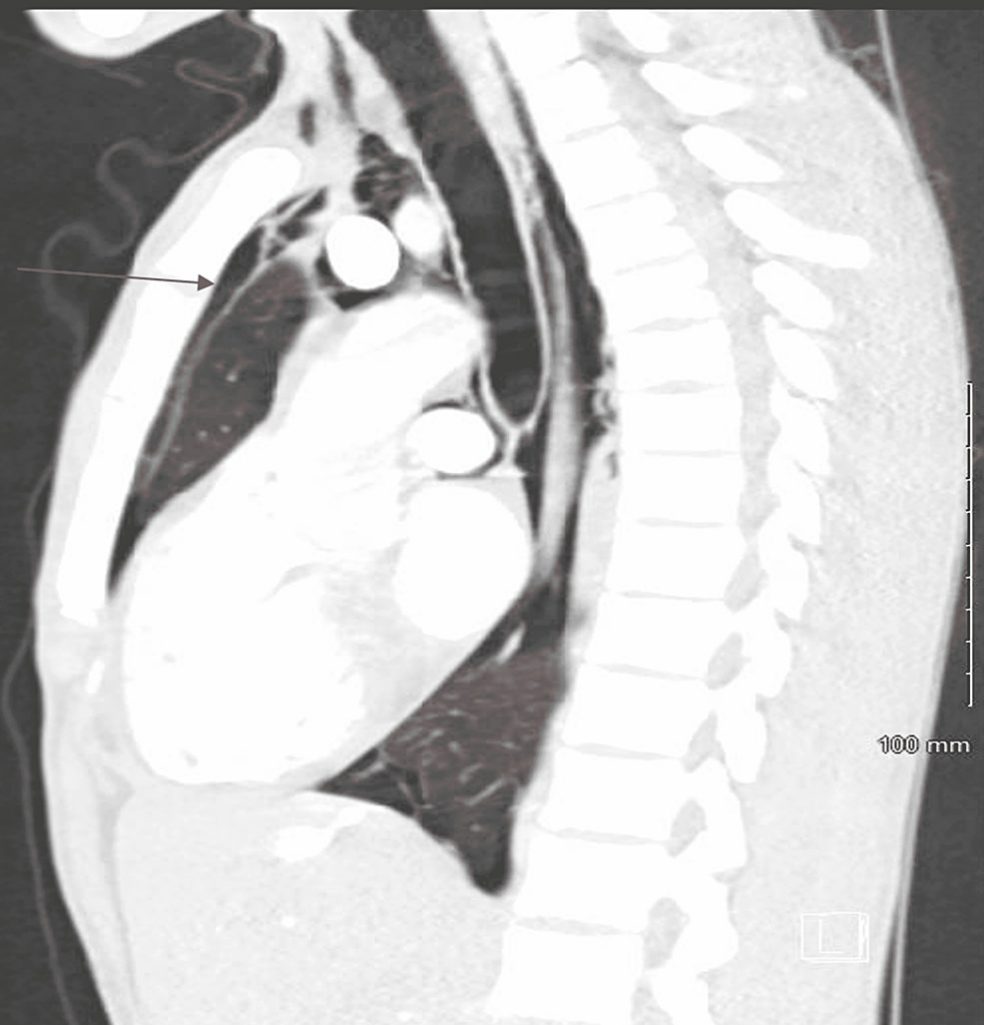
CT of the chest with contrast in sagittal plane showing pneumothorax as indicated by the arrow.

CT of the chest with contrast showed pneumomediastinum without fluid collection and small apical pneumothorax on the right side without tension pathology (Figures 3, 4).

**Figure 5:**
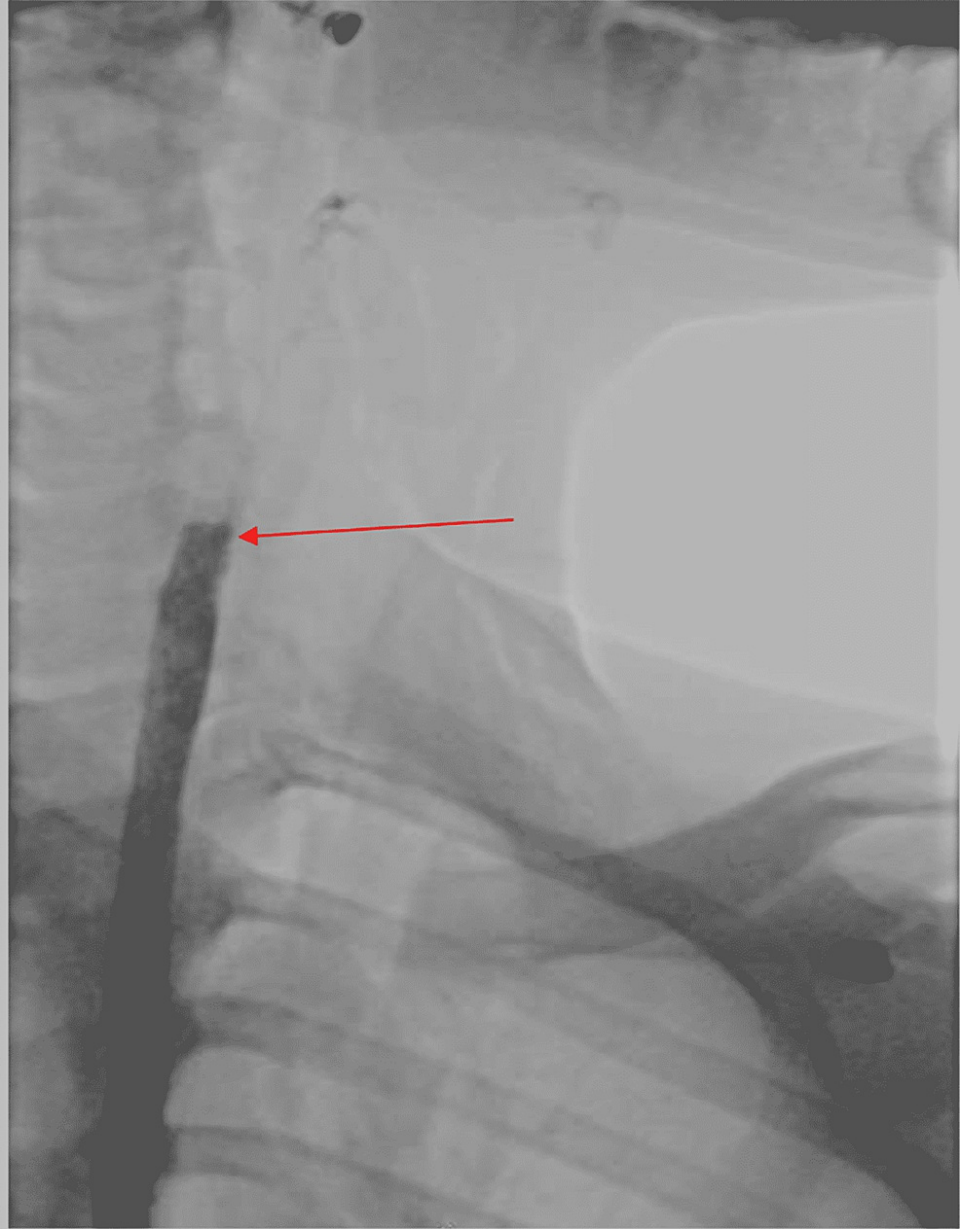
Barium esophagogram showing the normal contour of the esophagus without any evidence of irregularity or leak.

To rule out esophageal rupture, an esophagogram was performed, which revealed a normal contour of the esophagus without any evidence of irregularity or leak (Figure 5).

**Figure 6:**
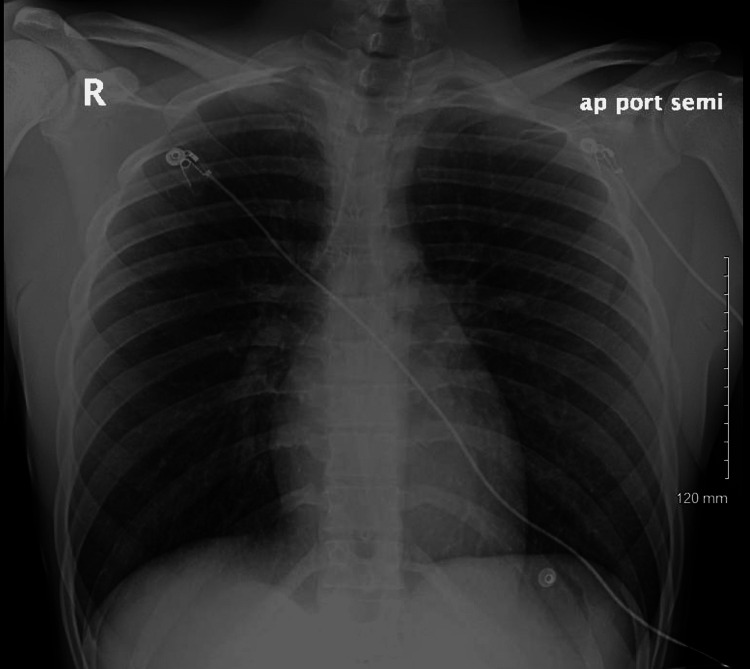
Chest radiograph in the anteroposterior view demonstrating no evidence of a pneumomediastinum or pneumothorax.

The patient was given normal saline boluses to correct his acute kidney injury. Cardiothoracic surgery advised against surgical intervention, and the patient was managed conservatively with oxygen support via nasal cannula, prophylactic piperacillin/tazobactam, antiemetics, proton pump inhibitor, and fluids, and was kept nil per oral initially. The patient remained hemodynamically stable for the duration of his hospitalization, and serial chest X-rays showed no advancement of pneumothorax or pneumomediastinum. He was discharged home on day four of hospitalization on antiemetics, and a proton pump inhibitor. He was cautioned against further cannabis use and was advised to follow up with psychiatry as an outpatient. Follow-up chest imaging performed six months after the initial presentation demonstrated complete resolution of pneumothorax and pneumomediastinum (Figure 6).

## Discussion

Due to cannabis' psychoactivity leading to its misuse potential and lack of safety data, the "Marihuana Tax Act of 1937" classified cannabis as a schedule 1 narcotic substance, which is a category that includes lysergic acid diethylamide (LSD), heroin, and methylenedioxy-methylamphetamine (MDMA) or more commonly known as ecstasy [4,5]. Several US states have legalized the recreational and/or medical use of marijuana, making it vital to investigate, analyze, and report the consequences of this formerly illegal and understudied substance [6]. In 1994, 35% of high school students considered cannabis a non-harmful substance, which has increased to 64% as surveyed in 2004. Both tobacco and cannabis smoke has been shown to include carcinogenic compounds, including phenol, nitrosamines, vinyl chloride, and polycyclic aromatic hydrocarbons, which have been associated with lung, oropharyngeal, and gonadal cancer. During a single cannabis smoking session, Wu et al. discovered that three times as much substance is transferred to the mouth as opposed to tobacco smoking, whereas one-third of the substance inhaled from cannabis smoke stayed in the lung tissues [7]. Compared to tobacco, marijuana smoking is related to a 66% yield in puff volume and a 33% rise in breathing depth and inhalation duration. Furthermore, as cannabis smokers hold their breath four times longer than cigarette smokers, smoking marijuana deposits three times as much tar in the lungs as smoking cigarettes [7]. Cyclic vomiting syndrome related to marijuana use, also known as cannabinoid hyperemesis syndrome, is defined by bouts of several episodes of vomiting with normal health intervals, correlation with chronic marijuana use, and remission after discontinuation of marijuana usage [8]. Breathing maneuvers associated with cannabis smoking, such as Valsalva and breath-holding, and repeated episodes of vomiting as observed in cannabinoid hyperemesis syndrome are believed to be one of the pathophysiologic reasons behind cannabis users' susceptibility to barotrauma, hence resulting in a pneumothorax. Both the toxic elements associated with marijuana inhalation and the barotrauma associated with breath-holding maneuvers may contribute to the development of pneumothorax in a patient who habitually smokes marijuana; however, additional research is necessary to demonstrate a causal association [2]. As a single episode of spontaneous pneumothorax places patients at significant risk for a recurrence, it is imperative that patients refrain from inhaling marijuana.

## Conclusions

Further investigation is essential to estimate the incidence of pulmonary pathology associated with marijuana smoking and associated bullous lung disease. Although some research has been done, further research is required to clarify the mechanism by which marijuana smoking affects pulmonary health.

## References

[1] Patel RS, Kamil SH, Bachu R (2020). Marijuana use and acute myocardial infarction: a systematic review of published cases in the literature. Trends Cardiovasc Med.

[2] Mishra R, Patel R, Khaja M (2017). Cannabis-induced bullous lung disease leading to pneumothorax: case report and literature review. Medicine (Baltimore).

[3] Aujayeb A, Donald C, Doe S (2012). Breath-holding in a marijuana smoker. Respir Med Case Rep.

[4] (2022). Drug Enforcement Administration. Drug scheduling. https://www.dea.gov/drug-information/drug-scheduling.

[5] Musto DF (1972). The Marihuana Tax Act of 1937. Arch Gen Psychiatry.

[6] Mead A (2017). The legal status of cannabis (marijuana) and cannabidiol (CBD) under U.S. law. Epilepsy Behav.

[7] Wu TC, Tashkin DP, Djahed B, Rose JE (1988). Pulmonary hazards of smoking marijuana as compared with tobacco. N Engl J Med.

[8] Sharma U (2018). Cannabis hyperemesis syndrome. BMJ Case Rep.

